# Low dose aspirin associated with greater bone mineral density in older adults

**DOI:** 10.1038/s41598-022-19315-0

**Published:** 2022-09-01

**Authors:** Hongzhan Liu, Xungang Xiao, Qiaojing Shi, Xianzhe Tang, Yun Tian

**Affiliations:** 1 Department of Joint Surgery, Hengyang Medical School, The Chenzhou Affiliated Hospital, University of South China, Hengyang, 421001 Hunan China; 2 Department of Joint Surgery, Chenzhou No.1 People’s Hospital, Chenzhou, 423000 Hunan China; 3 Department of Oncology, Affiliated Hospital of Xiangnan University, Chenzhou, 423000 Hunan China

**Keywords:** Health care, Medical research

## Abstract

The use of low-dose aspirin in older adults is increasing as is the prevalence of osteoporosis. Aspirin has been shown in numerous studies to affect bone metabolism. However, there is no clear link between low-dose aspirin use and bone mineral density (BMD). This study examined differences in bone mineral density between low-dose aspirin users and non-aspirin users in adults aged 50–80 years. We conducted a cross-sectional study of 15,560 participants who participated in the National Health and Nutrition Examination Survey (NHANES) 2017-March 2020. We used a multivariate logistic regression model to evaluate the relationship between low-dose aspirin and femoral neck BMD, femoral total BMD, intertrochanteric BMD, and the first lumbar vertebra BMD (L1 BMD) in patients aged 50 to 80 years. A total of 1208 (Group 1: femoral neck BMD, total femur BMD, and intertrochanter BMD) and 1228 (Group 2: L1 BMD) adults were included in this study. In both group 1 and group 2, BMD was higher in the low-dose aspirin group than in the non-aspirin group (Total femur BMD β = 0.019, 95% CI 0.004–0.034; Femoral neck BMD β = 0.017, 95% CI 0.002–0.032; Intertrochanter BMD β = 0.025, 95% CI 0.007–0.043; L1 BMD β = 0.026, 95% CI 0.006–0.046). In subgroup analyses stratified by gender, this positive association existed in both gender after adjusting for confounders. On subgroup analyses stratified by age, this positive association existed in three different age groups after adjusting for confounders. To test whether the effect of low-dose aspirin on BMD was affected by gender and age, the interaction P value was greater than 0.05. These findings from a human study looking into the relationship between low-dose aspirin use and BMD suggest that regular low-dose aspirin may be associated with a higher BMD. The association between low-dose aspirin and BMD did not differ by age group or gender.

## Introduction

Osteoporosis (OP) is a systemic bone metabolic disease in which bone loss exceeds bone formation, leading to reduced bone mass, bone microarchitecture deterioration, bone fragility, and fracture susceptibility^1^. Many endogenous and exogenous factors influence bone metabolism. Prostaglandin E2 (PGE2) is a precursor of inflammatory factors synthesized by arachidonic acid in the enzyme Cyclooxygenase (COX), which affects bone metabolism^2^. It is critical for osteoblast and bone tissue formation and may influence osteoclast formation by altering the RANKL-OPG axis^2–6^. Aspirin belonging to NSAIDs inhibits the synthesis of PGE2 by inhibiting COX, thus affecting bone metabolism^7^. Aspirin could either result in decreased or increased BMD, depending on the balance of prostaglandins in bone tissue^8^. Aspirin inhibited osteoclast formation by inhibiting the activation of NF-κB and MAPKS in RANKL-induced RAW264.7 cells, thereby increasing bone mineral density^9^. A recent meta-analysis by Barker et al. found that aspirin was associated with higher total hip and lumbar spine BMD in women and men^10^. However, aspirin, on the other hand, increases physiological nitric oxide (NO) production^11,12^, which may decrease osteoblasts and increase osteoclast activity, resulting in a reduction in BMD.

Osteoporosis and cardiovascular disease often coexist and our of major public health concern^13^. Aspirin is a cyclooxygenase inhibitor used mainly by the elderly to prevent cardiovascular disease^14^. Low-dose aspirin (75–100 mg/day orally) may be considered to prevent Atherosclerotic Cardiovascular Disease (ASCVD) in people aged 40 to 70 with no risk of bleeding^15^. Data from the 2010 U.S. National Health Interview Survey found that 19% of 27,157 subjects aged 18 years and older took aspirin^16^. Among current preventive aspirin users, 70% took low-dose aspirin (75–100 mg/day orally) daily^17^. There are about 2 million osteoporosis-related fractures annually, and the net cost to the U.S. Medicare system is $19 billion^18,19^. Clinical significance of osteoporosis is highlighted by the increasing prevalence of fragility fractures. Around 536,000 new brittle fractures occur annually in the U.K., including 79,000 hip fractures and 66,000 clinically diagnosed spinal fractures^20^. As measured by BMD, osteoporosis was defined at the bone mass level ^21^. Measurements at the hip and spine are typical areas for assessing and monitoring BMD are commonly used as an outcome measure in trials for about 30 years^22^. Given the widespread use of aspirin in older adults, it may be essential to ascertain whether it affects bone mineral density. This study aimed to investigate the association between low-dose aspirin use and bone mineral density among adults 50–80 years of age using the National Health and Nutrition Examination Survey (NHANES) data.

## Materials and methods

### Study population

Data for this study came from NHANES conducted between 2017 and March 2020, using a complex, multi-stage, hierarchical, clustered probability sample design to select a representative sample of civilians rather than a simple random sample based on the U.S. population^23^. The Ethics Review Committee of the National Center for Health Statistics approved all NHANES protocols and obtained written informed consent from all participants. The relevant guidelines and regulations performed all methods.

**Figure 1:**
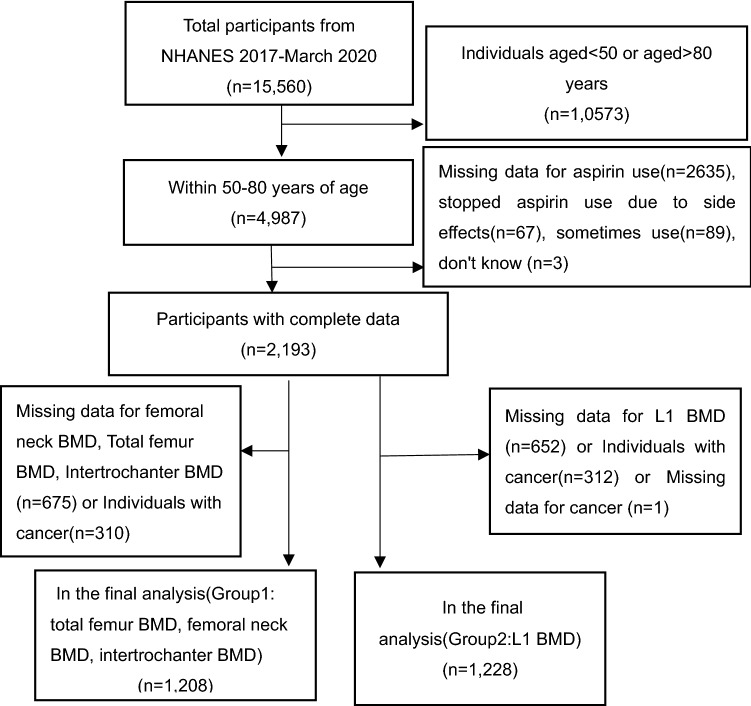
Flow chart of NHANES sample selection from March 2017 to March 2020.

A total of 15,560 participants were analyzed from 2017 to 2020 (Fig. 1). Excluding patients younger than 50 or older than 80 (n = 10,857) there were 4987 participants. 2794 people who were not eligible were excluded for the following reasons: Missing data for aspirin use (n = 2635), stopped aspirin use due to side effects (n = 67), sometimes using aspirin (n = 89), do not know (n = 3). Additionally, after excluding individuals with cancer and missing data for BMD there were 1208 patients with femoral BMD (group 1) and 1128 with L1 BMD (group 2). The National Center for Health Statistics’ Institutional Review Board reviewed and approved the survey protocols, and each participant signed a consent form voluntarily^24^.

### Evaluation of exposures

The exposure variables in this study were based on in-person home interviews with participants who reported aspirin use prophylaxis and followed recommendations for low-dose aspirin use. The questions to prevent aspirin use were asked at home by trained interviewers using a computer-assisted Personal Interview (CAPI) system. Questions ascertaining aspirin use, “Have you taken a daily low-dose aspirin as recommended by your doctor and other health care provider to prevent heart disease, stroke, or cancer?”. Categorical answers included “don’t know (n = 3), sometimes take aspirin (n = 89), stopped taking medication due to side effects (n = 67)” were excluded from the statistical analysis. The American college of cardiology foundation and the American heart association (ACC/AHA) defined the use of low-dose aspirin for preventive reasons as taking 75–100 mg orally daily^15^.

### Outcome

Association of aspirin use with femoral neck BMD, total femur BMD, intertrochanter BMD and L1 BMD, a measurement that has been used in the assessment and treatment of osteoporosis as a result of a clinical trial. All subjects in this study received a dual-energy X-ray absorptiometry (DXA) whole-body scan except for pregnant women, individuals who had used a radiographic contrast agent (such as dye or barium) within the past seven days; and individuals who weighed more than 208 kg (DXA table limit). A routine scan of the left hip was performed unless the participants reported a left hip fracture and had surgery on the left hip. In 2017–2018, the femur and spine scans were obtained using Apex 3.2 software on Hologic Discovery Type A Densimeter (Hologic, Inc., Bedford, Massachusetts). The femur and spine scans were taken on the Hologic Horizon Model A densimeter (Hologic, Inc., Bedford, Massachusetts) from March 2019 to March 2020. All scans obtained during March 2017–2020 were analyzed using APEX V4.0 (Hologic) software. Experts checked the scans for accuracy and consistency.

### Covariates

The categorical variables listed below were used in this study as covariates: gender, race, heart attack, vigorous work activity, education level, fractures, anti-osteoporotic, prednisone or cortisone, smoking status, and alcohol consumption. Standardized questionnaires were used to obtain the above variables^25^. Our analysis included successive covariables: age, BMI, Hba1c, the ratio of family income to poverty, C Reactive Protein (CRP), serum creatinine, and serum uric acid.

Race or ethnicity was categorized as non-Hispanic whites, non-Hispanic blacks, Mexican–American, Other Hispanic, and Other Race—Including Multi-Racial. Education level was categorized as Less than high school, high school, and more than high school. Less than high school was defined as Less than 9th grade and 9-11th grade (Includes 12th grade with no diploma). High school was defined as high school graduate/GED or equivalent. More than high school was defined as some college or AA (Associate of Arts) degree and college graduate or above. The ratio of family income to poverty was calculated by dividing total annual family (or individual) income by the poverty guidelines specific to the survey year^26^. Smoking status was categorized as Every day, Some days, not at all. Vigorous work activity was assessed via a history of participating in one of the following activities during the past week. This is based on responses to the question, “Does your job involve a high-intensity activity, such as lifting or lifting heavy objects, digging or construction work, for at least ten consecutive minutes, that causes a significant increase in breathing or heart rate?” Vigorous work activity was categorized as “yes” or “no” according to the participants’ answered. Alcohol consumption was categorized as never in the last year, nearly every day, 2 to 4 times a week, less than 2 times a week. This is based on responses to the question, “During the past 12 months, how often did you drink any alcoholic beverage?” Fracture history was categorized based on the question “Did the doctor tell you about any other fractures?” Categorize fractures as “yes” or “no”.

Consistency checking is built into CAPI systems to reduce data entry errors. CAPI also uses online help filters to help interviewers define critical terms in the questionnaire. After collection, interview data are reviewed by NHANES field office staff to ensure the accuracy and completeness of selected items. Interviewers were asked to conduct taped interviews, which were reviewed by National Center for Health Statistics (NCHS) staff and interviewers.

### Statistical analysis

Non-response and unequal selection probability samples were interpreted with weights. Data analysis in this study was conducted by the analysis guidelines prepared by the U.S. National Bureau of Statistics, using R version 3.4.3 (http://www.R-project.org) and filling in statistical software (http://www.empowerstats.com). 0.05 (P value) was defined as a significant level. A multivariate logistic regression model was used to evaluate the relationship between low-dose aspirin use status and femoral neck BMD, total femur BMD, intertrochanter BMD and L1 BMD. Following the Strengthening, the Reporting of Observational Studies in Epidemiology (STROBE) statement guidelines^27^, subgroup analyses stratified by age and gender were chosen, and interaction tests were run to make the data more useful. The relevant guidelines and regulations performed all methods. Three models were constructed: Model 1: no covariables are adjusted; Model 2: age and race or age and sex are adjusted; Model 3: all covariables are adjusted.

### Ethical statement

The Ethics Review Committee of the National Center for Health Statistics approved all NHANES protocols and obtained written informed consent from all participants. All methods were performed by the relevant guidelines and regulations.

## Results

According to the characteristics of the weighted sample shown in Table 1, the following are the characteristics of non-aspirin and low-dose aspirin users (group1: total femur BMD, femoral neck BMD, intertrochanter BMD group): compared with the non-aspirin group (n = 353), the low-dose aspirin group (n = 855) was older (65.7 ± 8.5 VS 63.0 ± 8.3 years P < 0.05). And total femur BMD was higher (0.925 ± 0.149 0.946 ± 0.167 P = 0.037). In the non-aspirin group, the proportion was 40.3% in females, 37.4% in the 50–60 years old group, 40.5% in the 60–70 years old group, and 22.0% in 70–80 years old group. Women accounted for 42.5% of the low-dose aspirin group. 27.7% of participants were 50–60 years old, 36.3% were 60–70, and 36.0% were 70–80 years old. Total femur BMD, intertrochanter BMD, BMI, Hba1c, heart attack, smoking status, fractures, and education level were significantly different between the two groups (P < 0.05).

**Table 1:** Weighted characteristics of study sample non-aspirin users and low-dose aspirin users (Group1: Total femur BMD, Femoral neck BMD, Intertrochanter BMD).

	Non-aspirin use (n = 353)	Low-dose aspirin use (n = 855)	P value
Total femur BMD (gm/cm^2^)	0.925 ± 0.149	0.946 ± 0.167	0.037
Femoral neck BMD (gm/cm^2^)	0.756 ± 0.132	0.774 ± 0.151	0.053
Intertrochanter BMD (gm/cm^2^)	1.099 ± 0.174	1.126 ± 0.198	0.030
BMI (kg/m^2^)	29.13 ± 5.15	30.03 ± 5.86	0.012
Hba1c (%)	6.01 ± 1.26	6.24 ± 1.08	0.002
Serum creatinine(mmol/L)	83.29 ± 23.64	83.62 ± 30.23	0.852
Serum uric acid (umol/L)	328.27 ± 77.46	329.13 ± 80.70	0.864
**Gender (%)**			0.464
Male	59.73	57.45	
Female	40.26	42.54	
Age (years)	63.07 ± 8.39	65.78 ± 8.51	< 0.001
**Age groups (%)**			< 0.001
50–60 years	37.44	27.67	
60–70 years	40.50	36.25	
70–80 years	22.04	36.07	
**Race (%)**			0.096
Mexican American	5.55	4.29	
Other Hispanic	8.79	5.23	
Non-Hispanic White	69.69	71.43	
Non-Hispanic Black	9.70	10.44	
Other race-including multi-racial	6.25	8.59	
**Heart attack (%)**			< 0.001
Yes	5.73	16.57	
No	94.26	83.38	
Recorded		0.04	
**Education level (%)**			0.011
Less than high school	10.11	11.77	
High school	27.41	35.96	
More than high school	62.47	52.21	
Recorded		0.03	
**Fractures (%)**			0.001
Yes	35.73	25.40	
No	64.19	74.47	
Recorded	0.07	0.12	
**Smoking status (%)**			0.00
Every day	16.04	10.18	
Some days	4.03	1.25	
Not at all	33.55	35.24	
Recorded	46.36	53.31	

Mean ± SD for continuous variables: P value was calculated by the weighted linear regression model.

Per cent for categorical variables: P value was calculated by weighted chi-square test. Weights are created in NHANES to account for the complex survey design (including oversampling), survey non-response, and post-stratification adjustment to match total population counts from the Census Bureau.

In Table 2, the following are the characteristics of non-aspirin and low-dose aspirin users (group2: L1BMD): compared with the non-aspirin group (n = 350), the low-dose aspirin group (n = 878) was older (65.8 ± 8.5 and 63.0 ± 8.4 years P < 0.05). In the non-aspirin group, the proportion was 41.8% in females, 58.2% in males, 38.6% in the 50–60 years old group, and 38.5% in the 60–70 years old group, and 22.9% in 70–80 years old group. Women accounted for 44.7% of the low-dose aspirin group, while men accounted for 55.3%. A total of 28.3% in the 50–60 years old group, 34.9% in the 60–70 years old group, and 36.8% in the 70–80 years old group. L1 BMD, BMI, Hba1c, Heart attack, smoking status, and education level were also significantly different between the two groups (P < 0.05).

**Table 2:** Weighted characteristics of study sample non-aspirin users and low-dose aspirin users (Group2: L1BMD).

	Non-aspirin use (n = 350)	Low-dose aspirin use (n = 878)	P value
L1 BMD (gm/cm^2^)	0.963 ± 0.159	1.006 ± 0.189	< 0.001
Age(years)	63.03 ± 8.46	65.72 ± 8.60	< 0.001
**Age groups (%)**			< 0.001
50–60 years	38.64	28.31	
60–70 years	38.48	34.88	
70–80 years	22.87	36.80	
BMI (kg/m^2^)	29.55 ± 5.55	30.87 ± 6.53	< 0.001
Hba1c (%)	6.07 ± 1.27	6.27 ± 1.11	0.008
Serum creatinine (mmol/L)	83.06 ± 22.27	84.18 ± 34.46	0.574
Serum uric acid (umol/L)	327.82 ± 76.09	331.71 ± 81.46	0.443
**Gender (%)**			0.353
Male	58.21	55.29	
Female	41.78	44.70	
**Race (%)**			0.198
Mexican American	5.48	4.70	
Other Hispanic	8.48	5.18	
Non-Hispanic White	68.64	70.71	
Non-Hispanic Black	10.39	10.43	
Other race-including multi-racial	6.99	8.96	
**Education level (%)**			0.002
Less than high school	10.31	12.22	
High school	26.81	35.59	
More than high school	62.87	52.18	
**Heart attack (%)**			< 0.001
Yes	5.46	15.98	
No	94.38	83.97	
Recorded	0.15	0.04	
**Fractures (%)**			0.085
Yes	34.20	27.81	
No	65.72	72.03	
Recorded	0.07	0.15	
**Smoking status (%)**			0.002
Every day	14.91	10.68	
Some days	4.11	1.30	
Not at all	32.93	35.38	
Recorded	48.03	52.63	

Mean ± SD for continuous variables: P value was calculated by the weighted linear regression model.

Per cent for categorical variables: P value was calculated by weighted chi-square test. Weights are created in NHANES to account for the complex survey design (including oversampling), survey non-response, and post-stratification adjustment to match total population counts from the Census Bureau.

Table 3 shows the gender-based stratified analysis and interaction tests. Table 3 shows the gender-based stratified analysis and interaction tests. Low-dose aspirin was positively associated with bone mineral density in both males (Total femur BMD β = 0.025, 95% CI 0.004–0.046; Femoral neck BMD β = 0.014, 95% CI − 0.006 to 0.035; Intertrochanter BMD β = 0.032, 95% CI 0.007–0.057; L1 BMD β = 0.041, 95% CI 0.013–0.069) and females (Total femur BMD β = 0.017, 95% CI − 0.004 to 0.038; Femoral neck BMD β = 0.021, 95% CI − 0.001 to 0.043; Intertrochanter BMD β = 0.021, 95% CI − 0.006 to 0.049; L1 BMD β = 0.005, 95% CI − 0.023 to 0.034), P interaction > 0.05, indicating that the effect of low-dose aspirin on BMD was not significantly different between genders.

**Table 3:** Subgroup analysis was stratified by gender.

	Total femur BMD (gm/cm^2^)	Femoral neck BMD (gm/cm^2^)	Intertrochanter BMD (gm/cm^2^)	L1 BMD (gm/cm^2^)
**Male**				
Non-aspirin users	Reference	Reference	Reference	Reference
Low-dose aspirin users	0.025 (0.004, 0.046) P = 0.0179	0.014 (− 0.006, 0.035) P = 0.1775	0.032 (0.007, 0.057) P = 0.0113	0.041 (0.013, 0.069) P = 0.0042
**Female**				
Non-aspirin users	Reference	Reference	Reference	Reference
Low-dose aspirin users	0.017 (− 0.004, 0.038) P = 0.1218	0.021 (− 0.001, 0.043) P = 0.0572	0.021 (− 0.006, 0.049) P = 0.1235	0.005 (− 0.023, 0.034) P = 0.7127
P interaction	P = 0.7746	P = 0.4587	P = 0.7612	P = 0.0882

Age, Race, Heart attack, Vigorous work activity, BMI, Hba1c, Education level, Ratio of family income to poverty, Fractures, Anti-osteoporotic, Prednisone or Cortisone, HS C-Reactive Protein, Smoking status, Serum Creatinine, Serum Uric acid, Alcohol consumption were adjusted.

Table 4 shows the stratified analysis and interaction tests by age. Stratified analysis by age showed that the positive association between low-dose aspirin use and bone mineral density remained in three different age groups: 50–60 years (Total femur BMD β = 0.020, 95% CI − 0.010 to 0.049; Femoral neck BMD β = 0.026, 95% CI − 0.004 to 0.056; Intertrochanter BMD β = 0.028, 95% CI − 0.008 to 0.064; L1 BMD β = 0.026, 95% CI − 0.007 to 0.059), 60–70 years (Total femur BMD β = 0.037, 95% CI 0.013–0.061; Femoral neck BMD β = 0.014, 95% CI − 0.011 to 0.040; Intertrochanter BMD β = 0.047, 95% CI 0.018–0.077; L1 BMD β = 0.029, 95% CI − 0.013 to 0.062), 70–80 years (Total femur BMD β = 0.010, 95% CI − 0.009 to 0.039; Femoral neck BMD β = 0.013, 95% CI − 0.013 to 0.039; Intertrochanter BMD β = 0.008, 95% CI − 0.028 to 0.044; L1 BMD β = 0.011, 95% CI − 0.030 to 0.052). The interaction p value of low-dose aspirin on bone mineral density was greater than 0.05 in 50–60 years old, 60–70 years old and 70–80 years old, indicating that there was no significant difference in the effect of low-dose aspirin on bone mineral density in different age groups.

**Table 4:** Subgroup analyses stratified by age.

	Total femur BMD (gm/cm^2^)	Femoral neck BMD (gm/cm^2^)	Intertrochanter BMD (gm/cm^2^)	L1 BMD (gm/cm^2^)
**50–60 years**				
Non-aspirin users	Reference	Reference	Reference	Reference
Low-dose aspirin users	0.020 (− 0.010, 0.049) P = 0.1907	0.026 (− 0.004, 0.056) P = 0.0931	0.028 (− 0.008, 0.064) P = 0.1289	0.026 (− 0.007, 0.059) P = 0.1190
**60–70 years**				
Non-aspirin users	Reference	Reference	Reference	Reference
Low-dose aspirin users	0.037 (0.013, 0.061) P = 0.0024	0.014 (− 0.011, 0.040) P = 0.2614	0.047 (0.018, 0.077) P = 0.0015	0.029 (− 0.004, 0.062) P = 0.0887
**70–80 years**				
Non-aspirin users	Reference	Reference	Reference	Reference
Low-dose aspirin users	0.010 (− 0.019, 0.039) P = 0.4979	0.013 (− 0.013, 0.039) P = 0.3352	0.008 (− 0.028, 0.044) P = 0.6663	0.011 (− 0.030, 0.052) P = 0.5989
P interaction	P = 0.3347	P = 0.7506	P = 0.2308	P = 0.7528

Gender, Race, Heart attack, Vigorous work activity, BMI, Hba1c, Education level, Ratio of family income to poverty, Fractures, Anti-osteoporotic, Prednisone or Cortisone, HS C-Reactive Protein, Smoking status, Serum Creatinine, Serum Uric acid, Alcohol consumption were adjusted.

Total femur BMD β = 0.019, 95% CI 0.004–0.034; Femoral neck BMD β = 0.017, 95% CI 0.002–0.032; Intertrochanter BMD β = 0.025, 95% CI 0.007–0.043; L1 BMD β = 0.026, 95% CI 0.006–0.046.

Table 5 shows the results of multiple regression analysis. In the unadjusted model 1, low-dose aspirin use was positively associated with bone mineral density (Total femur BMD β = 0.021, 95% CI 0.001–0.041; Femoral neck BMD β = 0.018, 95% CI − 0.000 to 0.036; Intertrochanter BMD β = 0.026, 95% CI 0.003–0.050; L1 BMD β = 0.043, 95% CI 0.021–0.066). After adjusting for confounders, this positive association persisted in model 2 (adjusted for age, gender, and race) (Total femur BMD β = 0.041, 95% CI 0.024–0.057; Femoral neck BMD β = 0.034, 95% CI 0.019 to 0.050; Intertrochanter BMD β = 0.048, 95% CI 0.029–0.067; L1 BMD β = 0.051, 95% CI 0.030–0.072) and model 3 (adjusted for all covariates) (Total femur BMD β = 0.019, 95% CI 0.004–0.034; Femoral neck BMD β = 0.017, 95% CI 0.002–0.032; Intertrochanter BMD β = 0.025, 95% CI 0.007–0.043; L1 BMD β = 0.026, 95% CI 0.006–0.046 P < 0.05). On average, low-dose aspirin users had 0.019 g/cm^2^ higher total femoral BMD, 0.017 g/cm^2^ higher femoral neck BMD, 0.025 g/cm^2^ higher intertrochanteric BMD, and 0.026 g/cm^2^ higher L1 BMD than non-aspirin users.

**Table 5:** Associations between low-dose aspirin use and bone mineral density.

	Model1, β (95% CI, P)	Model2, β (95% CI, P)	Model3, β (95% CI, P)
**Total femur BMD (gm/cm**^**2**^**)**			
Non-aspirin use	Reference	Reference	Reference
Low-dose aspirin use	0.021 (0.001, 0.041) P = 0.03786	0.041 (0.024, 0.057) P < 0.00001	0.019 (0.004, 0.034) P = 0.01137
**Femoral neck BMD (gm/cm**^**2**^**)**			
Non-aspirin use	Reference	Reference	Reference
Low-dose aspirin use	0.018 (− 0.000, 0.036) P = 0.05388	0.034 (0.019, 0.050) P = 0.00001	0.017 (0.002, 0.032) P = 0.02779
**Intertrochanter BMD (gm/cm**^**2**^**)**			
Non-aspirin use	Reference	Reference	Reference
Low-dose aspirin use	0.026 (0.003, 0.050) P = 0.03016	0.048 (0.029, 0.067) P < 0.00001	0.025 (0.007, 0.043) P = 0.00743
**L1 BMD (gm/cm**^**2**^**)**			
Non-aspirin use	Reference	Reference	Reference
Low-dose aspirin use	0.043 (0.021, 0.066) P = 0.00017	0.051 (0.030, 0.072) P < 0.00001	0.026 (0.006, 0.046) P = 0.00941

Model 1 no covariates were adjusted.

Model 2 age, gender, and race were adjusted.

Model 3 Gender, Age, Race, Heart attack, Vigorous work activity, BMI, Hba1c, Education level, Ratio of family income to poverty, Fractures, Anti-osteoporotic, Prednisone or Cortisone, HS C-Reactive Protein, Smoking status, Serum Creatinine, Serum Uric acid, Alcohol consumption were adjusted.

## Discussion

This cross-sectional study investigated the relationships of low-dose aspirin use with total femur BMD, femoral neck BMD, intertrochanter BMD, and L1BMD. In summary, our results mainly show significantly higher total femur BMD, intertrochanter BMD, and L1 BMD in low-dose aspirin users compared to non-aspirin users in the general US population and significantly greater femoral neck BMD but only after multivariate adjustment. Stratified analysis and interaction test showed that the effect of low-dose aspirin on bone mineral density had no significant difference among different age groups and genders.

In Tables 1 and 2, aspirin users have higher BMI and higher Hba1c, which may be because that obese patients or diabetic patients are more likely to suffer from cardiovascular diseases and need aspirin. The number of fractures in patients who took aspirin was significantly lower than in those who did not. This could mean that people with broken bones are more likely to have cardiovascular disease or that aspirin use might reduce the risk of fractures. This requires further study, the lower prevalence of daily smoking among aspirin users does not mean that smokers are less likely to develop the cardiovascular disease but rather that daily smokers are likely to be younger and have a lower risk of cardiovascular disease. In Table 3, in stratified analysis by gender, total femur BMD, intertrochanter BMD, and L1BMD were significantly higher in male aspirin users than in non-aspirin users. However, the P values of the interaction were all greater than 0.05, which may indicate no difference in the effect of aspirin on BMD between the genders. In Table 4, in age stratification, although the total femur BMD and intertrochanter BMD of patients aged 60 to 70 who used aspirin were higher than that of those who did not use aspirin, all P values of interaction were more significant than 0.05, which may indicate that there was no difference in the effect of aspirin on BMD among age groups. In Table 5, in the multiple regression model, after adjusting variables in model 2 and Model 3, total femur BMD, femoral neck BMD, intertrochanter BMD, and L1BMD of aspirin users was significantly higher than that of patients of non-aspirin users (P < 0.05), and femoral neck BMD of aspirin users in model 1 was higher than that of non-aspirin users not significant (P = 0.053). Possibly due to the influence of sample size, it is expected that increasing sample size will significantly increase bone mineral density.

The majority of low-dose aspirin users are elderly, who have higher rates of cardiovascular disease morbidity and mortality^28,29^. In addition to its antiplatelet activity, aspirin may also affect oxidative stress, vascular inflammation, and insulin sensitivity^30–35^. Recent studies have shown that low-dose aspirin can affect bone metabolism^10,14,36–41^. But these research results are contradictory and controversial.

Early results suggest that aspirin can improve BMD and bone structure in young rat models of osteoporosis^42–44^. These results come from animal studies, and there is no definitive evidence in humans. Another study observed that, after adjusting for confounders, women who took aspirin five to seven times a week had higher bone mineral density in the hips and spine than women who did not take aspirin^40^. The findings are difficult to generalize to other populations because the subjects were all white women^40^. Carbone et al. found significant increases in systemic BMD in subjects using aspirin alone and aspirin plus selective nonsteroidal anti-inflammatory drugs relative to COX2^39^. Only two ethnic groups were represented in the study: 43.1% African American and 56.9% Caucasian. The average age of the study participants was 73.6 years (19–70 years). One study showed that long-term use of low-dose aspirin was not associated with decreased bone mineral density in the general population^45^. Individuals over 65 were excluded from the study, even though it was a large population study with multiple confounders. However, due to the large elderly population of low-dose aspirin users, the study’s sample size was limited to 47 low-dose aspirin users, so the results are not representative of the general population. Despite a growing number of observational studies, there have been no randomized controlled trials of the effect of aspirin treatment on fracture risk in humans, and the relationship between aspirin use and bone mineral density is contradictory^37,39,40,46^.

This research has several limitations. As the study is cross-sectional, it cannot describe changes in BMD with aspirin over time and causality in the relationship cannot be inferred. Secondly, we had no information on the duration of aspirin therapy or the exact dose. We were unable to a relationship of aspirin dose to BMD because this study lacked information on the exact dose of aspirin. Thirdly we only measured BMD, but other measures that capture bone quality and microarchitecture, including Computerized tomography (CT), were unavailable, or bone turnover markers were^47,48^. Fourthly, while we adjusted for the presence of cardiovascular disease, we were not able to specifically take account of medications like thiazide diuretics and statins which are associated with higher BMD.

On the other hand, to better summarize the U.S. population, this study included a representative multiracial population sample with a large sample size that could be further analyzed by subgroup. In addition, we adjusted for multiple potential confounding factors, including heart attack, vigorous work activity, education level, fractures, anti-osteoporotic, prednisone or Cortisone, HS C-Reactive Protein, smoking status, serum creatinine, serum uric acid, and alcohol consumption.

Overall, our research suggests that low dose aspirin may improve bone mineral density irrespective of age or gender. However, as studies are cross-sectional further research to assess longitudinal changes in BMD in low-dose aspirin users should be conducted. Additionally, randomized controlled trials would be required to prove causality.

## Data Availability

All data generated or analyzed during this study are included in this published article.
